# Systems intervention to promote colon cancer screening in safety net settings: protocol for a community-based participatory randomized controlled trial

**DOI:** 10.1186/1748-5908-8-58

**Published:** 2013-06-03

**Authors:** Aimee S James, Veronica Richardson, Jean S Wang, Enola K Proctor, Graham A Colditz

**Affiliations:** 1 Division of Public Health Sciences, Department of Surgery, Washington University School of Medicine, 660 S. Euclid Ave, Campus Box 8100, St. Louis, MO 63110, USA; 2 Grace Hill Health Centers, Quality Improvement and Corporate Compliance, 1717 Biddle Street, St. Louis, MO 63106, USA; 3 Division of Gastroenterology, Department of Medicine, Washington University School of Medicine, 660 S. Euclid Avenue, Campus Box 8124, Saint Louis, MO 63110, USA; 4 Brown School, Washington University, St. Louis, MO 63110, USA

**Keywords:** Colon cancer, Healthcare disparities, Screening, Randomized controlled trial, Intervention studies, Multi-level intervention, Implementation strategy, Community-based participatory research

## Abstract

**Background:**

Colorectal cancer is a leading cause of cancer mortality. Screening can be effective but is underutilized. System- or multi-level interventions could be effective at increasing screening, but most have been implemented and evaluated in higher-resource settings such as health maintenance organizations. Given the disparities evident for colorectal cancer and the potential for screening to improve outcomes, there is a need to expand this work to include diverse settings, including those who treat economically disadvantaged patients. This paper describes the study protocol for a trial designed to increase colorectal cancer screening in those ‘safety-net’ health centers that serve underinsured and uninsured patients. This trial was designed and is being implemented using a community-based participatory approach.

**Methods/design:**

We developed a practical clinical cluster-randomized controlled trial. We will recruit 16 community health centers to this trial. This systems-level intervention consists of a menu of evidence-based implementation strategies for increasing colorectal cancer screening. Health centers in the intervention arm then collaborate with the study team to tailor strategies to their own setting in order to maximize fit and acceptability. Data are collected at the organizational level through interviews, and at the provider and patient levels through surveys. Patients complete a survey about their healthcare and screening utilization at baseline, six months, and twelve months.

**Outcomes:**

The primary outcome is colorectal cancer screening by patient self-report, supplemented by a chart-audit in a subsample of patients. Implementation outcomes informed by the Reach, Efficacy/Effectiveness, Adoption, Implementation, and Maintenance (RE-AIM) conceptual framework will be measured at patient, provider, and practice levels.

**Discussion:**

Our study is one of the first to integrate community participatory strategies to a randomized controlled trial in a healthcare setting. The multi-level approach will support the ability of the intervention to affect screening through multiple avenues. The participatory approach will strengthen the chance that implementation strategies will be maintained after study completion and, supports external validity by increasing health center interest and willingness to participate.

**Trial registration:**

NCT01299493

## Background

Colorectal cancer (CRC) is a leading cause of cancer death among men and women in the United States [1]. Many CRC deaths could be avoided through prevention and early detection activities; early stage CRC is associated with excellent survival [1]. Routine screening for CRC is recommended starting at age 50 years for adults at average-risk; several different screening tests have been found effective and are recommended at varying intervals [2,3]. CRC is unique among cancers, because screening is associated not only with early detection, but with prevention [4]. Cancers can be averted through the identification and removal of precancerous polyps. Yet, colorectal screening is underutilized; at least 40% of age-eligible U.S. adults are not adherent to current screening guidelines [5,6]. This rate is lower than many other cancer screenings.

There are disparities in screening and survival from CRC. Individuals who are lower income, underinsured or uninsured, or are from a racial/ethnic minority are less likely to get screened and more likely to be diagnosed at later stages [1,7]. Uninsured patients fare worse than their insured counterparts even when adjusting for stage at diagnosis [7-12]. Given that CRC is a leading cancer diagnosis and disparately experienced, and that effective prevention and early detection tools reduce mortality by one-half but are under-utilized in routine primary care settings [13,14], it is important to investigate the feasibility and effectiveness of strategies to increase screening.

Interventions to increase CRC rates that are focused on individual level factors have had only minimal to moderate success in getting patients screened. While individual-level strategies can be effective and have promise, systems- or multi-level approaches have the potential to reach a wider swath of the patient population and thus may have more overall impact [15-18]. Such approaches can set the stage to increase the likelihood of patient-provider discussion about CRC screening as well as increase the likelihood that screening is referred and completed. Most interventions have been tested in structured and well-resourced settings [19-22]. In response to a Request for Applications (CA 09–032) from the National Cancer Institute requesting a randomized trial using Community-Based Participatory Research (CBPR) methods, we met with our Colon Cancer Community Advisory Board (CAB) to identify needs and elicit preferences and suggestions for study design and implementation. Our objective in this study is to conduct a practical clinical trial [23-27] to evaluate the viability and effectiveness of evidence-based implementation strategies in community health centers serving populations disparately affected by CRC. To achieve our stated aims, we are developing, implementing, and evaluating a systems-level intervention aimed at increasing CRC screening. The long-term goal is to reduce CRC disparities by developing sustainable and disseminable implementation approaches that will effectively promote informed decisions about CRC screening across a variety of settings, particularly those considered underserved. Our protocol is described below.

## Methods

This delayed start cluster randomized controlled study is designed as a ‘practical clinical trial’ [23-28]. As such, it is designed to test the impact of an evidence-based intervention under ‘real world’ conditions. Using a rolling recruitment, community health centers (CHCs) are randomized to either intervention or comparison (*i.e.*, delayed intervention). The primary outcome is CRC screening rates as assessed by patient self-report. Other outcomes, such as implementation fidelity and reach, will be evaluated according to the Reach, Efficacy/Effectiveness, Adoption, Implementation, and Maintenance (RE-AIM) conceptual framework [29-33]. This study underwent review by the Washington University Human Research Protection Office (IRB Protocol #201110005).

### Role of community-based participatory research in this study

Based on the RFA and the input from our CAB, we designed this research to follow established CBPR principles, including acknowledging the community; fostering co-learning and capacity building for all; building on strengths and resources within the community; integrating and achieving a balance of all partners; facilitating collaborative, equitable partnership in all phases of research; a focus on local relevance and determinants of health; involving partners and systems development in a cyclical and iterative process; disseminating finding and knowledge gained to all partners and involving all partners in the dissemination process; and planning for a long-term process and commitment [34,35]. Key aspects of CBPR incorporated in this trial include collaborative partnership during design, development, implementation, and evaluation, an emphasis on local relevance, and a commitment to long-term relationships and sustainability of interventions. As such, the early outcomes and formative work involved in this research inform the procedures used in the latter activities. Partnered activities and decisions occur throughout the trial. Our cancer center’s Program for the Elimination of Cancer Disparities had an existing network of community and clinical partners who formed our CAB and helped facilitate the integration of CBPR into the proposal development and planning.

### Theoretical framework

In addition to applying CBPR principles, another goal of our study was to incorporate a multi-level approach that acknowledged practical factors related to implementation across settings. We chose the RE-AIM framework [29-33] as the guide for our measures selection and analysis plan. RE-AIM describes different components of dissemination research and is useful for translating research to practice and for conceptualizing the external validity of a trial. The following RE-AIM concepts are incorporated into our measures and analyses: reach and representativeness, differences in implementation between CHCs, adoption of the trial and of the various evidence-based implementation strategies by CHCs and providers, effectiveness of the overall intervention in increasing screening, and maintenance of resultant strategy changes after the end of data collection. The study aims to reach patient, healthcare providers, and systems that affect care and care provision.

### Setting and participants

#### Community health centers

To be eligible, CHCs must serve mostly Medicaid, uninsured, or lower-income patients; be willing to be randomized to intervention or comparison, and willing to allow the research team access to CHC managers/directors, patients, and providers. Sites (n = 16) are recruited on a rolling basis. We chose this approach in order to better serve the sites’ timetables and availability, and to tap on their enthusiasm for improving quality of care at their sites. Recruiting all sites at the beginning of the trial would force some CHCs to wait before the study started at their site, risking dropout due to administrative turnover or emerging or competing needs of the CHC.

There are over 30 CHCs in our metropolitan region. CHCs were initially identified from amongst our existing community partners. Next, we created a list of federally-qualified CHCs and other known CHCs in our targeted region, and approached them to assess interest. Where possible, we start with a known contact; in other sites, the health center Chief Executive Officer or Chief Medical Officer is identified from websites. In all cases, potential sites are emailed an Institutional Review Board-approved invitation letter and study information sheet. Follow-up is conducted by the study team via email and telephone. In most cases, a combination of email, telephone, and in-person contacts will be made before CHCs formally agree to participate in the trial. With each CHC, we conduct an organizational assessment upon entry and exit into the survey. Additionally, CHC providers will answer anonymous surveys at the start/end of the study to assess their awareness and perception of the trial. Organizational and provider surveys will help us evaluate the RE-AIM constructs of reach, adoption, implementation, and maintenance. We will also be able to better characterize the representativeness (external validity) of our participating sites.

#### Patients

Although the intervention is delivered at the practice-level, primary outcomes of CRC screening will be assessed via self-report from patients. Patient surveys will inform effectiveness and implementation (for example, if educational materials at the clinic reached patients).

A random sample of patients will be recruited from each participating CHC for the survey. Inclusion criteria include: age ≥49, English-speaking, having contact information listed in the medical record, and been seen at the CHC within the last two years. Patients are recruited by mailed invitation letter from the health center about the study and giving them the opportunity to opt out. Study team members will follow-up with all patients who do not opt out. We will aim to collect baseline data from 100 to 110 respondents per clinic where possible. With 70 to 75% retention by 12 months, this will result in adequate power to detect intervention effects. We recognize that some sites may have smaller patient populations and that retaining participants over the course of the 12-month study may be harder when working with underserved populations whose contact information may change during the study period. However, restricting our sample to only health centers with large or stable patient populations would limit external validity.

The study team considered using chart reviews as the primary outcome. However, given the strength of support for the validity of self-reported CRC screening [36-39], and the difficulty of chart reviews in low-resource settings where electronic medical records may be harder to search, requiring chart reviews would be a barrier to CHC participation and potentially decrease external validity. We will use a chart audit on a subset of sites for further verification of self-report in this population (see section entitled Data collection – Patient-level data).

#### Intervention

The intervention consists of a menu of evidence-based strategies for increasing CRC screening. There are several evidence-based systems interventions to promote CRC screening in primary care, but few [20] have been tested in underserved populations or in real-world settings. We selected the primary strategies that have evidence for their effectiveness based on the CDC Community Guide to Preventive Services [40,41] and the American Cancer Society/National Colorectal Cancer Roundtable Toolkit for physicians [42,43]: routine patient reminders, provider reminders, provider feedback, and structural changes. These changes can reach the multiple levels of patient care including systems, providers, and patients. CHCs randomized to the intervention arm will be presented with the standard ‘menu’ of implementation strategies and will be able to select the strategies they wish to implement for increasing screening. We will partner with individual sites to tailor these strategies into specific interventions that are compatible with their site and perceived as offering advantage over current practices. Comparison CHCs will be offered the menu of strategies at the end of the trial after data collection has ended. All sites receive access to patient education materials.

We chose this ‘menu’ approach for several reasons: data indicate that healthcare providers are more likely to adhere to an intervention if they helped design and select it [44]; this approach allows sites to decide which strategies are feasible, relevant, and sustainable in their context (*e.g.*, provider feedback might be difficult in a CHC without electronic records or with basic electronic systems; patient reminders are challenging if patient contact information is not reliable); offering choice enhances generalizability and representativeness by encouraging more wide-spread participation and buy-in by CHCs; and important to our CBPR approach, our community and clinical partners strongly felt that CHCs need to have an active role in selecting the intervention, rather than being ‘told what to do’ by the researchers.

Once decision makers and stakeholders at a HC are identified, the study team will meet with them to discuss their site’s challenges (and current practices) regarding CRC screening, and discuss the general main implementation strategies (reminders, feedback, etc.). Through discussion and consensus, the research team will then develop a menu tailored to each site that is then presented back to the same group for their final decisions. Additional personnel from the CHC will be consulted as needed (*e.g.*, Information Technology officers if a strategy involves the electronic medical record; health center managers if strategies are directed at patients, etc.).

#### Data collection

Our primary outcome will be CRC screening (percent of patients up-to-date on CRC screening per the U.S. Preventive Services Guidelines [45]) based on a survey of patients age-eligible for screening at baseline, six months, and twelve months. As we will describe, data are also collected at the provider- and practice-level.

#### Patient-level data

The baseline survey will include: demographics, healthcare utilization, medical home, cancer screening, literacy [46], behavior intentions, barriers to CRC screening [47-49], and knowledge about colon cancer. Standard measures from national surveys are used as available. The six-month and 12-month surveys include self-reported CRC screening, healthcare utilization, and awareness of screening or educational efforts. CHCs may add site-specific questions if they wish.

#### Recruitment for the patient survey

Procedures for patient recruitment will be finalized in collaboration with the participating sites. At most sites, the research team will work with the CHC to randomly select eligible patients and mail letters of invitation to potential participants. Letters will have an opt-out option and will be followed up by telephone. We expect that some sites will not want to mail letters out due to privacy concerns. In those cases, participants will be recruited in person at the participating health center. We will track the type of recruitment used and evaluate any potential impact or differences.

#### General procedures for follow-up data collection

Follow-up data will be collected via telephone survey. These call attempts will be made on multiple days at different times of the day. After approximately five call attempts with no answer, or two voicemail messages, the study staff will mail a letter stating that we are attempting contact. About a week after the letter is mailed, we will attempt one more ‘round’ of calls. Calling will continue until attempts to reach the participant have been exhausted (determination that there are no working telephone numbers), participants have completed the survey or declined participation, or the participant is more than three weeks past their survey due-date.

#### Chart review

Chart review procedures will be worked out with each CHC, who can opt in or out of this review. We aim to recruit at least 10 of our 16 CHCs to participate in the chart review, recognizing that such a procedure is onerous for CHCs without a flexible electronic medical record system. Chart review data will be used to supplement and support the self-report findings. We aim to review 74 charts per CHC.

#### Provider-level data

To understand how the implementation strategies (and the study) are perceived by the people who work at the health center and to assess whether strategies directed at providers actually reached them, we will also conduct brief, anonymous surveys of health center employees. Surveys will be distributed at the start of the study and post-intervention. In general, these questions will address RE-AIM principles, including implementation, perceived maintenance, feasibility, acceptability, but we may add questions to the post-intervention survey based on experiences and feedback during implementation. To maximize response, this survey will be brief (one page) and can be mailed in a pre-paid envelope, or faxed to a secure fax machine. For example, if a CHC chose to develop provider reminders in their electronic record, the post-survey would ask if the provider had seen the reminder and what they thought about it.

#### Practice-level data

We will conduct an organizational assessment by interviewing clinic administrators in order to address reach, implementation, and maintenance of the intervention and implementation strategies. Interviews will be audiotaped and transcribed, when the interviewee consents. This semi-structured interview will cover: patient characteristics, current efforts to promote CRC screening if any, current CRC screening rates, current efforts to promote other preventive care or disease management, perceived need for change, preferred changes, and barriers to implementation. The research team will work with the CHC data manager, if necessary, to access some of this information. We will ask our initial contact at the health center to recommend other persons with whom we should speak, such as other level managers, chief operating officers, medical directors, quality improvement personnel, or opinion leaders within the practice. Data, when presented, will be de-identified (both to individual and to health center). Any characteristics that could be used to identify the administrator or the health center/health system will be masked or aggregated. Data will be used to inform the intervention development, but may be analyzed later (*e.g.*, to look at characteristics of CHCs that did or did not change screening rates). We will aim to conduct at least two interviews per participating site.

#### Retention of health centers and survey respondents

We will use multiple evidence-based methods to maximize retention. We do not anticipate site-level attrition once a CHC is enrolled, because CHCs are entitled to pick and choose which strategies they wish to implement and the data collection burden on them (organizational assessment and exit interview) is designed to be minimal. However, the participatory process can be burdensome and time-consuming, or perceived burden could deter sites from participation. Our procedures are designed to balance fidelity to CBPR with reducing ‘what we ask’ from participating sites. CHCs are reimbursed financially for all phases of the study, though this does not truly capture the time they spend in a participatory approach.

Attrition among patient participants is a potential limitation. We will apply best practices for retention [50-53] and collect complete contact information at enrollment (name, postal address. and at least two telephone numbers). Participants will be asked to list a secondary contact person as a locator. Differential attrition in the patient survey by study arm is unlikely because the intervention is at the center-level and sites are randomized.

We expect some attrition amongst health center employees, more so due to turnover within CHCs than because of the study. Our post-survey will ask how long the respondent has been at the health center, but because these surveys are not identifiable and not linked, we will not know whether the same people answered a staff survey at pre and post evaluation.

#### Outcomes and analysis

Our analysis will examine rates of being up-to-date on CRC screening, accounting for the clustered nature of the data. We will also assess which CHCs were reached by the study (and how representative they are of area CHCs), reach of the intervention within a CHC (who was ‘touched’ by the intervention, which strategies were adopted, implementation of strategies, and maintenance of the strategies by analysis of our organizational assessments, staff surveys, and qualitative feedback.

#### Power

This study was powered based on patient self-report of screening. With16 CHCs (n = 74 participants per cluster) and intra-class correlation at 0.04, we will have 80% power to detect a 15% point difference in screening between intervention and comparison. Prevalence rates are estimated based on our experiences with similar patient populations, local BRFSS data, and published screening rates. Intervention effects were estimated from the rough average of published percent increases in screening for our potential strategies. These strategies resulted in a range of 12 to 18 percentage point differences [54-64]. If intra-class correlation is higher but the impact is greater, we still maintain at least 80% power with this sample.

#### Analysis

Descriptive analysis will be used to characterize reach, external validity, adoption of strategies, and fidelity to implementation. Effectiveness of the intervention in increasing CRC screening will focus on self-reported patient completion of CRC screening at 12 months post-baseline. Statistical analysis will quantify the intervention effect at the cluster (CHC) and individual (patient) levels. For the individual-level analysis, we will use a generalized linear mixed model, adjusting for individual-level covariates, and cluster-level covariates, and accounting for the clustered nature of the data. Confounders will be identified as those variables that might influence the outcome and their association will be tested in a bivariate model. Variables that are significantly associated with the outcome will be included as potential covariates in the final adjusted model.

#### Trial status

The trial is currently ongoing. The first set of health centers have been recruited and patient data collection has begun.

## Discussion

Increasingly, researchers are trying to balance randomized controlled research with community-based research aimed at decreasing disparities. Our research design is aimed at testing the effect of an implementation intervention to increase colorectal cancer screening, and at the same time assess the viability (and challenge) of translating evidence-based strategies into diverse settings. The implementation of interventions at the practice-level should mean that patients at the CHC have equal chance to be exposed to an intervention if and when they come for an appointment; other approaches that require patient uptake might disproportionately reach select groups of patients.

Our approach to health center recruitment will allow us to examine whether settings are representative, including examining reach and adoption (two key elements of the RE-AIM theoretical framework) by tracking how many settings were approached, how many participated, and reasons for non-participation [65-68]. The assessment of participation and reasons for non-participation is a critical advancement toward scientific rigor.

Our decision for allowing a ‘menu’ of implementation strategies balances the ability to test intervention effects with a respect for the different contexts and preferences of each health center. We recognize that this approach is both a strength and a challenge in the current study. The challenge in our ‘menu’ design is that it will be difficult to know which strategy had impact, because CHCs can choose as few or as many as they want. However, our main question is whether the intervention approach of offering a menu of strategies is effective. Identifying the specific effect size of each individual strategy is secondary to the question of whether the overall approach can increase screening. We will carefully track organizational characteristics as well as which strategies are adopted and implemented by which CHCs. We plan to explore the differential effectiveness of strategies if there is enough variation. For these reasons, the menu approach strengthens our study and better informs both the literature and prevention practice.

Efforts at reducing disparities and increasing use of appropriate and recommended cancer screening must address the varying contexts in which primary care occurs and the many barriers to screening at the patient, provider, and clinic levels. Practical trials that address external validity, and strive to understand how interventions may (or may not) work in real-world settings are critical to reducing and ultimately eliminating disparities in cancer screening and survival [65-68].

## Abbreviations

CRC: Colorectal cancer; CHC: Community health center.

## Competing interests

The authors declared that they have no competing interest.

## Authors’ contributions

ASJ conceived the study and its design and led the writing of the manuscript. VR provided insight into developing studies at CHCs and procedures for working with CHCs. JW contributed to the design and selection of outcome measurements. EP advised on the design of the study, the theoretical framework, and the selection of implementation measures. GAC helped conceive the study and helped draft the manuscript. GAC and AJ helped obtained funding. All authors helped revise the manuscript and reviewed the final version. All authors read and approved the final manuscript.
